# Bone-conditioned medium contributes to initiation and progression of osteogenesis by exhibiting synergistic TGF-β1/BMP-2 activity

**DOI:** 10.1038/s41368-018-0021-2

**Published:** 2018-06-12

**Authors:** Maria B. Asparuhova, Jordi Caballé-Serrano, Daniel Buser, Vivianne Chappuis

**Affiliations:** 1 Laboratory of Oral Cell Biology, School of Dental Medicine, University of Bern, Bern, Switzerland; 2 Department of Oral Surgery and Stomatology, School of Dental Medicine, University of Bern, Bern, Switzerland; 3 Department of Oral and Maxillofacial Surgery, School of Dental Medicine, Universitat Internacional de Catalunya, Barcelona, Spain

## Abstract

Guided bone regeneration (GBR) often utilizes a combination of autologous bone grafts, deproteinized bovine bone mineral (DBBM), and collagen membranes. DBBM and collagen membranes pre-coated with bone-conditioned medium (BCM) extracted from locally harvested autologous bone chips have shown great regenerative potential in GBR. However, the underlying molecular mechanism remains largely unknown. Here, we investigated the composition of BCM and its activity on the osteogenic potential of mesenchymal stromal cells. We detected a fast and significant (*P* < 0.001) release of transforming growth factor-β1 (TGF-β1) from autologous bone within 10 min versus a delayed bone morphogenetic protein-2 (BMP-2) release from 40 min onwards. BCMs harvested within short time periods (10, 20, or 40 min), corresponding to the time of a typical surgical procedure, significantly increased the proliferative activity and collagen matrix production of BCM-treated cells. Long-term (1, 3, or 6 days)-extracted BCMs promoted the later stages of osteoblast differentiation and maturation. Short-term-extracted BCMs, in which TGF-β1 but no BMP-2 was detected, reduced the expression of the late differentiation marker osteocalcin. However, when both growth factors were present simultaneously in the BCM, no inhibitory effects on osteoblast differentiation were observed, suggesting a synergistic TGF-β1/BMP-2 activity. Consequently, in cells that were co-stimulated with recombinant TGF-β1 and BMP-2, we showed a significant stimulatory and dose-dependent effect of TGF-β1 on BMP-2-induced osteoblast differentiation due to prolonged BMP signaling and reduced expression of the BMP-2 antagonist noggin. Altogether, our data provide new insights into the molecular mechanisms underlying the favorable outcome from GBR procedures using BCM, derived from autologous bone grafts.

## Regenerative therapy: A cell culture medium that promotes bone formation

‘Bone-conditioned medium’ could improve oral bone regeneration therapy by promoting the proliferation and maturation of bone-forming cells. Building on recent research demonstrating the benefits of using cell culture medium prepared with bone chips (BCM) in such treatments, researchers led by Maria Asparuhova of the University of Bern, Switzerland, set out to elucidate the medium’s mechanisms. The team found that BCM incubated with bone chips for short periods—as little as ten minutes—contained heightened levels of signaling protein TGF-β1, which enhanced mouse bone marrow cell proliferation while downregulating maturation. BCM incubated for longer periods also generated increased levels of another protein, BMP-2, which boosted the maturation of bone-forming cells. This study reveals a sequential role of these two factors in oral bone development, and the potential physiological actions of BCM when used in regenerative therapies.

## Introduction

Despite the increasing number of new bone-grafting substitutes, autografts remain the gold standard for bone augmentation and reconstruction in oral, maxillofacial and orthopedic surgery due to their excellent and cost-effective combination of biological and mechanical properties.^1–3^ Autologous bone is the only clinically available bone graft source that contains viable osteogenic precursor cells (osteogenicity), releases growth factors capable of inducing new bone formation (osteoinduction), and provides a scaffold for the ingrowth of new blood vessels and the migration of osteoprogenitor cells (osteoconduction).^4^ The combination of collagen membranes with autologous bone and a superficial layer of deprotenized bovine bone mineral (DBBM) is a widely used guided bone regeneration (GBR) technique,^5,6^ which bears little risk of recession of the facial mucosa and sustains the long-term stability of the augmented volume.^2,7,8^ Graft consolidation depends on the orchestrated activation of numerous growth factors in both the host and the graft. However, a precise characterization of the factors released by bone autografts over time and their contribution to the bone-forming process remains lacking.

Recent research from our laboratory aimed to discover the molecular mechanisms that underlie the favorable long-term results from bone augmentation procedures using autologous bone chips in combination with a bone substitute. The harvesting technique significantly influences the survival of bone cells contained within the autograft,^9^ and subsequently alters the release of osteoinductive growth factors.^10^ Furthermore, a 24-hour extraction of untreated bone chips with cell culture medium had the potential to affect a variety of cell types implicated in graft consolidation.^11,12^ This so-called bone-conditioned medium (BCM) induces osteoclastogenesis in bone marrow cultures^13,14^, and improves oral fibroblast cell activity through transforming growth factor (TGF)-β1 signaling.^15–17^ Moreover, collagen membranes rapidly adsorb the TGF-β1 activity contained in BCM, provoking changes in the gene expression pattern of oral fibroblasts grown on the membranes.^18^ Thus, pre-coating DBBM and collagen membranes with biologically active BCM that is extracted from locally harvested autologous bone chips during the surgical procedure has great clinical potential.

In addition to TGF-β, bone formation is regulated by growth factors such as Bone morphogenic protein (BMP)-2, 4, 5, 6, 7, and 9.^19^ A short-term expression of BMP-2 is sufficient to irreversibly induce osteogenesis.^20^ Thus, the goal of the present study is to analyze the TGF-β1 and BMP-2 protein release from autologous bone into BCM that is harvested for short periods (minutes) corresponding to the time of a typical surgical procedure, as well as the protein release after extended periods of time corresponding to the early days after the augmentation procedure occurred. The study further aimed to investigate the osteogenic response induced by BCM in the mesenchymal stromal line, ST2, thus providing insights into the complexity of bone matrix dynamics and the clinical potential of BCM. We hypothesized that BCM harvested within minutes might be sufficiently potent to exert a positive effect on the osteogenic properties of ST2 cells.

## Results

### Release of TGF-β1 and BMP-2 from cortical bone chips over time

**Fig. 1:**
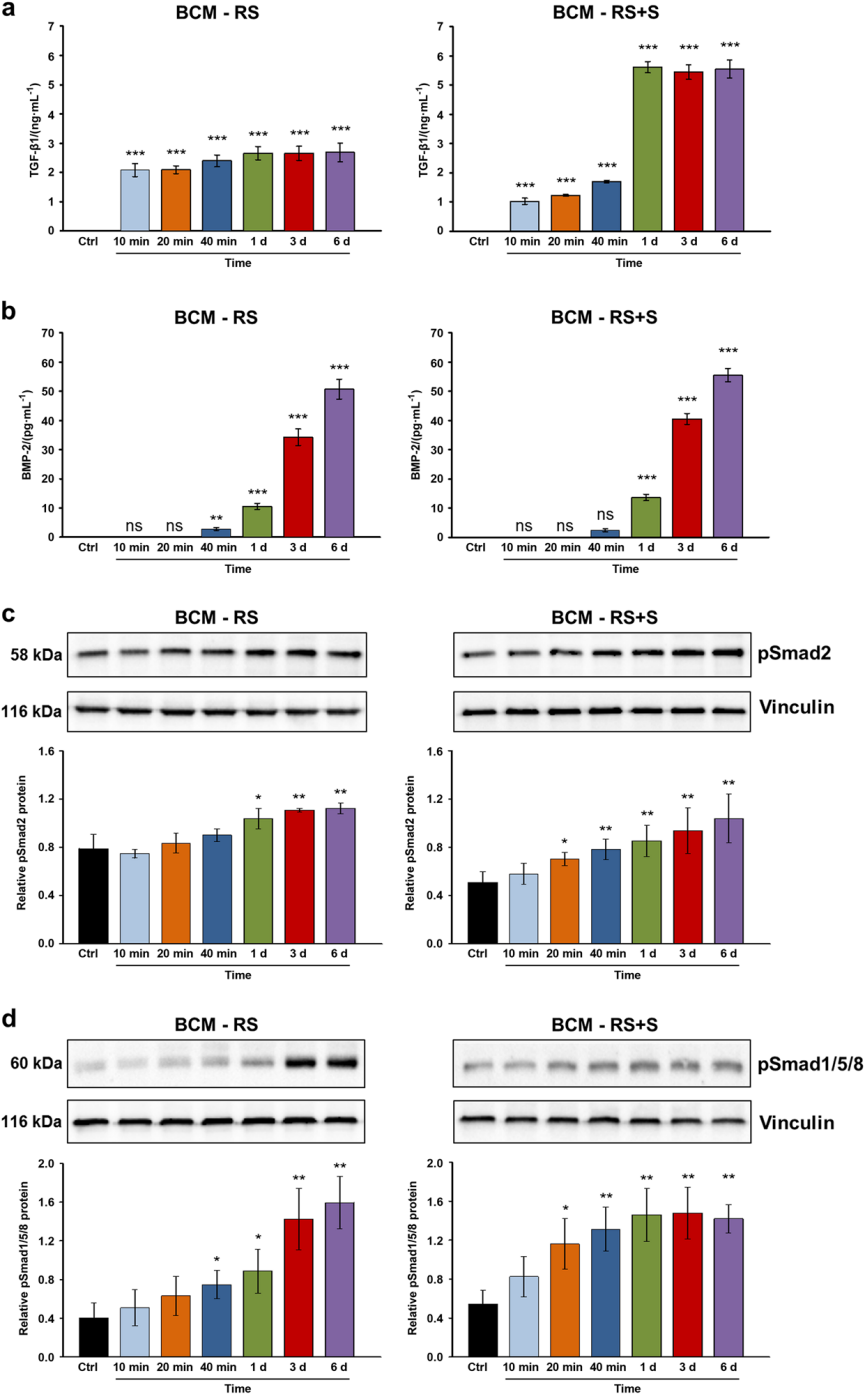
Release of TGF-β1 and BMP-2 proteins from the cortical bone and the induction of Smad signaling in mesenchymal stromal ST2 cells. **a**, **b** ELISA quantification of TGF-β1 (**a**) and BMP-2 (**b**) proteins contained in bone-conditioned medium extracted from the cortical bone chips with either Ringer’s solution (BCM-RS) or Ringer’s solution mixed with autologous serum (BCM-RS+S) at a 1:1 ratio. The two types of media were collected at 10, 20, and 40 min, as well as at 1, 3, and 6 d. Controls (ctrl) represent RS or RS+S, not containing bone particles (controls exhibited no detectable levels of the two proteins tested). Mean±standard deviations represent three independent BCM preparations, and significant differences to the respective controls (***P < 0.001, **P < 0.01, ns non-significant) are shown. **c**, **d** BCM induces enhanced phosphorylation of TGF-β1- and BMP-2-specific R-Smads in mesenchymal stromal ST2 cells. Immunoblot analyses of phospho-Smad2 (pSmad2) (**c**) and phospho-Smad1/5/8 (pSmad1/5/8) (**d**) proteins in whole-cell extracts from ST2 cells treated with BCM-RS or BCM-RS+S used in **a**, **b**. The bar charts represent densitometric quantification of the immunoblots. pSmad2 and pSmad1/5/8 levels are normalized to vinculin loading controls. Data represent mean±SD obtained from three independent experiments. Significant differences to the control cells treated with RS or RS+S (**P < 0.01, *P < 0.05) are shown

Bone chips extracted for various time periods showed very fast release kinetics for TGF-β1, compared to BMP-2 (Fig. 1a, b). Significant quantities of TGF-β1 (2.1 ng·mL^−1^, *P* < 0.001) were measured in BCM prepared with Ringer’s solution (RS) within 10 min (Fig. 1a, BCM-RS). The initial release (within minutes) of TGF-β1 into BCM prepared with a 1:1 mixture of Ringer’s solution and autologous serum (RS+S) (Fig. 1a, BCM-RS+S) was significant, but lower than the release into BCM-RS, most likely due to differences in the osmotic pressure generated by the two diluents. Whereas, no significantly higher quantities of TGF-β1 were detected in BCM-RS at time points longer than 10 min, the yields measured in BCM-RS+S prepared over 1, 3, and 6 d were over twofold higher (*P* < 0.001) than the amounts extracted at the same time points in RS. In contrast to TGF-β1, BMP-2 was not detected in BCM within 10 and 20 min in either of the two diluents (Fig. 1b). However, significant and increasing amounts (between 10 and 55 pg·mL^−1^, *P* < 0.001) of this growth factor were released from bone chips between 1 and 6 days.

### BCM induces enhanced phosphorylation of TGF-β1- and BMP-2-specific R-Smads in mesenchymal stromal ST2 cells

To investigate whether the quantities of TGF-β1 and BMP-2 released from bone chips within different time periods are sufficient to activate TGF-β and BMP signaling, respectively, we analyzed the phosphorylation of TGF-β1- and BMP-2-specific receptor-regulated Smad proteins (R-Smads) in BCM-treated ST2 cells (Fig. 1c, d). Smad2 responds to TGF-β1, whereas most BMPs activate Smad1/5/8 as their R-Smads. Compared to the basal levels of phospho-Smad2 detected in control RS-treated ST2 cells, the phosphorylation of Smad2 was significantly increased in cells treated with BCM-RS prepared over 1, 3, and 6 days (Fig. 1c, BCM-RS). BCM-RS+S appeared to be even more potent, since the preparation made within 20 min was already able to induce significant Smad2 phosphorylation (*P* < 0.05), compared to the basal phospho-Smad2 levels in the control cells (Fig. 1c, BCM-RS+S). Compared to control cells, significant phosphorylation of Smad1/5/8 was observed in cells treated with BCM-RS extracted from 40 min onwards as well as in cells treated with BCM-RS+S extracted from 20 min onwards (Fig. 1d).

### BCM harvested within short time periods induces proliferation of ST2 cells

**Fig. 2:**
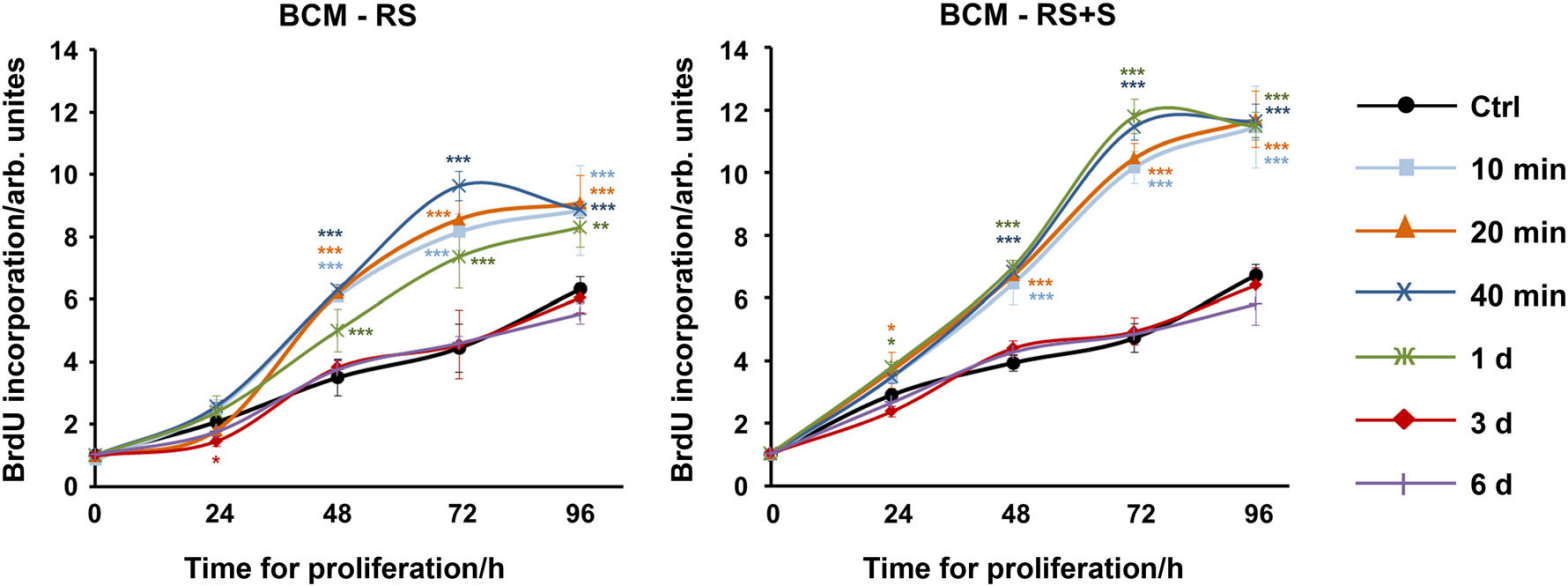
BCM extracted for short time periods induces proliferation of ST2 cells. Proliferation rates of ST2 cells treated with BCM-RS or BCM-RS+S, which were extracted for different time periods as described in Fig. 1a, b, were assessed by BrdU incorporation into newly synthesized DNA immediately after plating (0 h), as well as at 24, 48, 72, and 96 h. Experimental values were normalized to the values of control (ctrl) cells treated with RS or RS+S at time point 0. Mean± standard deviations represent four independent experiments, and significant differences to the control cells (***P < 0.001, **P < 0.01, *P < 0.05) are shown

Osteogenesis involves chemotaxis and proliferation of osteoblast precursors, differentiation to the mature osteoblast phenotype with the synthesis of extracellular matrix proteins, and mineralization of the resulting matrix. We first assessed the proliferative ability of ST2 cells treated with different BCM preparations (Fig. 2). Compared to control cells, ST2 cells treated with BCMs extracted for 10, 20, 40 min, or 1 d in each of the two diluents showed a significant increase in 5-bromo-20-deoxyuridine (BrdU) uptake into newly synthesized DNA until they reached confluence. In contrast, ST2 cells treated with BCMs extracted over 3 or 6 days behaved like the control cells. Thus, BCMs harvested for short periods (up to 1 day) strongly stimulated the proliferation of osteoprogenitors.

### BCM induces osteoblast differentiation of ST2 cells

To assess the osteogenic potential of BCM, we monitored the expression of genes encoding bone matrix proteins such as collagen type I (Col1a1 and Col1a2) and osteopontin (also known as secreted phosphoprotein 1, Spp1), as well as genes encoding the differentiation markers, runt-related transcription factor 2 (Runx2), osteocalcin (or bone gamma-carboxyglutamate protein 2, Bglap2), bone sialoprotein (or integrin-binding sialoprotein, Ibsp), and alkaline phosphatase (Alpl) in BCM-treated ST2 cells.

**Fig. 3:**
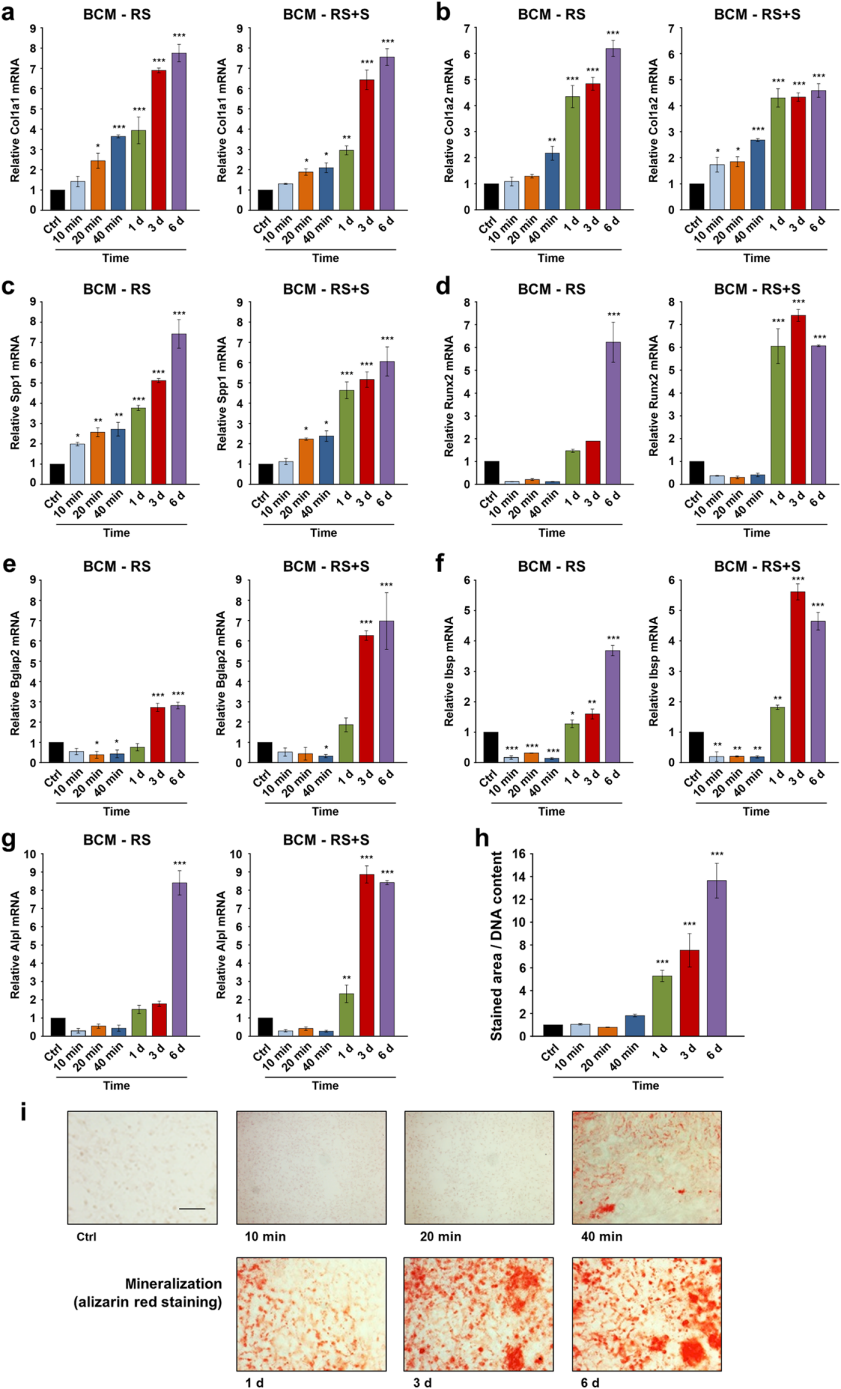
BCM induces osteoblast differentiation of ST2 cells. **a**–**g** Effect of BCM-RS or BCM-RS+S extracted for different time periods, as described in Fig. 1a, b, on Col1a1 (**a**), Col1a2 (**b**), Spp1 (**c**), Runx2 (**d**), Bglap2 (**e**), Ibsp (**f**), and Alpl (**g**) mRNA levels. ST2 cells were treated with 20% BCM-containing osteogenic media for 24 h before total RNA was extracted and analyzed by qRT-PCR. Values normalized to Gapdh are expressed relative to the values of control (ctrl) cells treated with 20% RS- or RS+S-containing osteogenic media. Data represent mean±SD obtained from three independent experiments. Significant differences to the respective control (***P < 0.001, **P < 0.01, *P < 0.05) are shown. **h**, **i** Effect of the BCM-RS preparations on the mineral deposition capacity of ST2 cells. Cells were treated with 20% BCM-containing osteogenic media for 14 days before extracellular matrix mineralization was analyzed by alizarin red staining. Cells cultured under the same conditions in 20% RS-containing osteogenic media were used as controls (ctrl). Mineral deposition was assessed and quantified by measuring the stained area using the Fiji distribution of ImageJ (**h**). Values normalized to DNA content are expressed relative to the values of control cells. Data represent mean± standard deviations obtained from three independent experiments. Significant differences to the control (***P < 0.001) are shown. Representative images of the staining in each of the experimental groups are shown (**i**). Scale bar=500 µm

BCMs made within short (10, 20, or 40 min) and long (1, 3, or 6 d) time periods in each of the two diluents (RS or RS+S) caused a continuous and significant increase in Col1a1, Col1a2, and Spp1 mRNAs above the expression levels detected in control cells treated with the diluents alone (Fig. 3a–c). This suggests a continuous accumulation in the BCM of factors released from the autografts that substantially influence the early stages of differentiation, namely, the production of matrix onto which the mineral is deposited.

Next, Runx2, Bglap2, Ibsp, and Alpl were generally upregulated in cells treated with BCMs extracted over days with a trend of higher potency exhibited by the BCM-RS+S compared to the BCM-RS preparations, most likely due to the complex composition of serum present in the former (Fig. 3d–g). In contrast, all four differentiation markers showed decreased mRNA levels in cells treated with BCMs that were extracted for minutes in each of the two diluents, compared to control cells. The downregulation was most significant for the late differentiation markers Bglap2 and Ibsp (Fig. 3e, f), suggesting an inhibitory effect of BCM extracted for minutes on osteoblast maturation.

Finally, we assessed the mineral deposition capacity of BCM-treated ST2 cells (Fig. 3h, i). In agreement with the gene expression analysis and similar to control cells grown in the absence of BCM, BCMs made within minutes did not induce any significant matrix mineralization. In contrast, mineral deposition was strongly and continuously enhanced more than five-fold (*P* < 0.001) in cells treated with BCMs extracted over 1, 3, and 6 days.

### TGF-β1 exhibits a stimulatory and dose-dependent effect on BMP-2-induced osteoblast differentiation

**Fig. 4:**
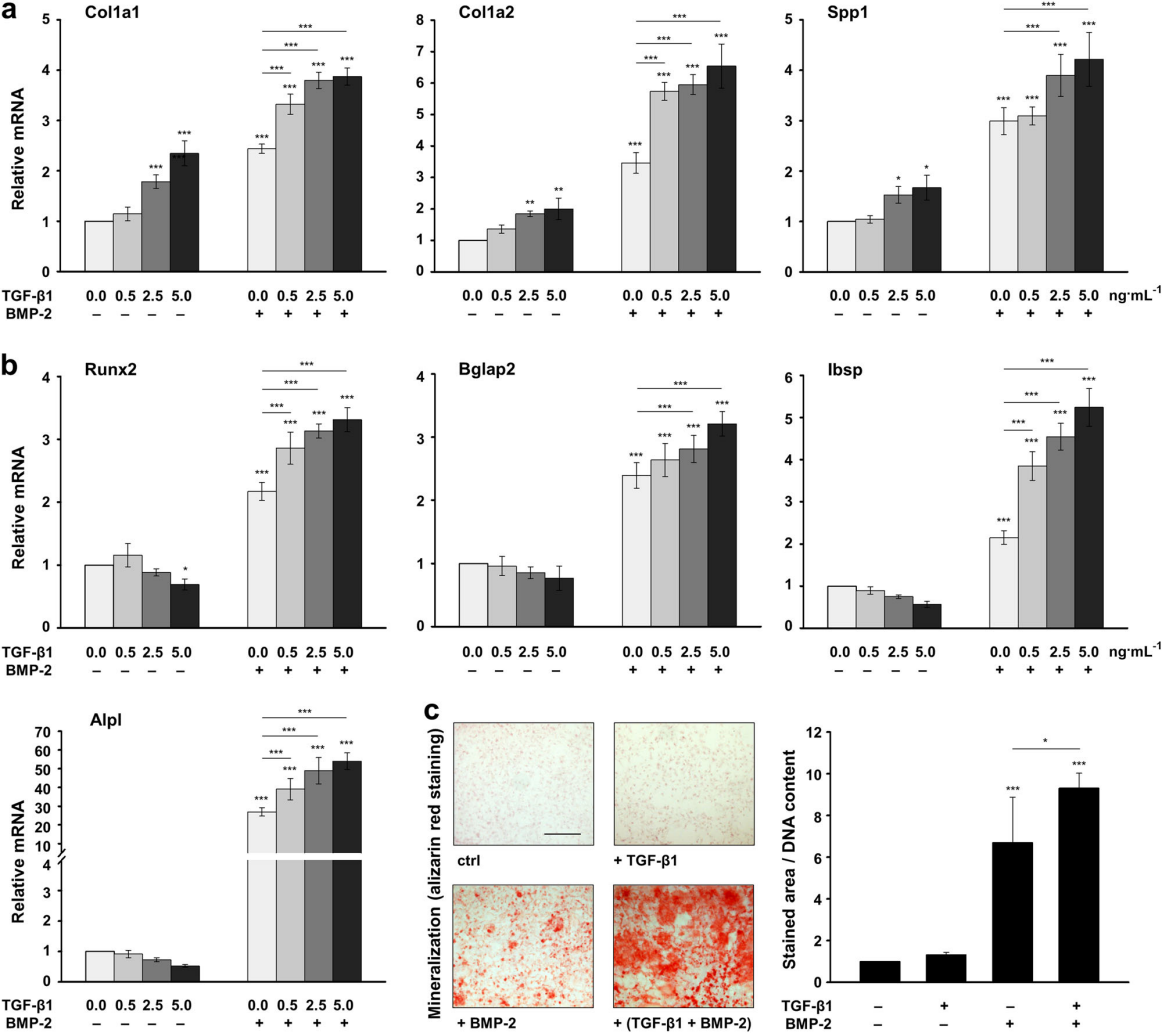
TGF-β1 exhibits a stimulatory and dose-dependent effect on BMP-2-induced osteoblast differentiation. **a**, **b** Effect of recombinant TGF-β1 and BMP-2 on the expression of osteoblast-specific genes in ST2 cells. Cells were treated with increasing concentrations (0.0, 0.5, 2.5, and 5.0 ng·mL^-1^) of TGF-β1 in the absence (−) or presence (+) of 100 ng·mL^-1^ BMP-2 for 24 h before total RNA was extracted. Expression of Col1a1, Col1a2, and Spp1 mRNAs (**a**), as well as Runx2, Bglap2, Ibsp, and Alpl mRNAs (**b**) was analyzed by qRT-PCR. Values normalized to Gapdh are expressed relative to the values of untreated cells. Mean± standard deviations were obtained from three independent experiments, and significant differences to either untreated cells or cells treated with BMP-2 alone (***P < 0.001, **P < 0.01, *P < 0.05) are shown. **c** Effect of the recombinant TGF-β1 and BMP-2 on the mineral deposition capacity of ST2 cells. Cells were treated with TGF-β1 (5 ng·mL^-1^), BMP-2 (100 ng·mL^-1^), or both growth factors for 4 d, and were subsequently grown under osteogenic culture conditions for 10 additional days before extracellular matrix mineralization was assessed by alizarin red staining. Representative images are shown. Scale bar=500 µm. Mineral deposition capacity was quantified by measuring the stained area using the Fiji distribution of ImageJ. Values normalized to the DNA content are expressed relative to the values of untreated control cells (ctrl). Data and statistical significance are expressed as in **a**, **b**

As shown above, BCM harvested for minutes induces genes encoding bone matrix proteins, but does not contribute to matrix mineralization, whereas BCMs prepared over days contribute to the progression of osteogenesis. These differential actions observed for BCMs containing TGF-β1 but no detectable BMP-2 levels versus BCMs containing both proteins simultaneously imply an intriguing crosstalk between the two growth factors. To study the combined effect of TGF-β1 and BMP-2 on osteoblast differentiation, ST2 cells were treated with increasing concentrations of recombinant TGF-β1 in the absence or presence of recombinant BMP-2 (Fig. 4). Similar to the effect seen in cells treated with short-term extracted BCMs, increasing concentrations of TGF-β1 applied in the absence of BMP-2 caused significant and dose-dependent increases of Col1a1, Col1a2, and Spp1 mRNAs (Fig. 4a), but a slight decrease of Runx2, Bglap2, Ibsp, and Alpl mRNAs (Fig. 4b), compared to the respective expression levels in untreated cells. As expected, the application of BMP-2 alone caused a significant upregulation of all osteoblast-specific mRNAs above the expression levels seen in untreated cells (Fig. 4a, b). Furthermore, a dose-dependent induction of all tested genes was observed when TGF-β1 was applied together with BMP-2.

Next, we assessed the mineralization capacity of growth factor-treated ST2 cells (Fig. 4c). As expected, mineralization was stimulated by BMP-2 but not TGF-β1. However, when the two growth factors were applied concomitantly, TGF-β1 significantly (*P* < 0.05) enhanced the BMP-2-induced deposition of mineralized matrix by ST2 cells.

Together, these results demonstrate that TGF-β1 exhibits a stimulatory and dose-dependent effect on the BMP-2-induced differentiation of ST2 cells towards the osteoblast lineage.

### TGF-β1 prolongs BMP-2 signaling and reduces the expression of the BMP-2 antagonist, noggin

**Fig. 5:**
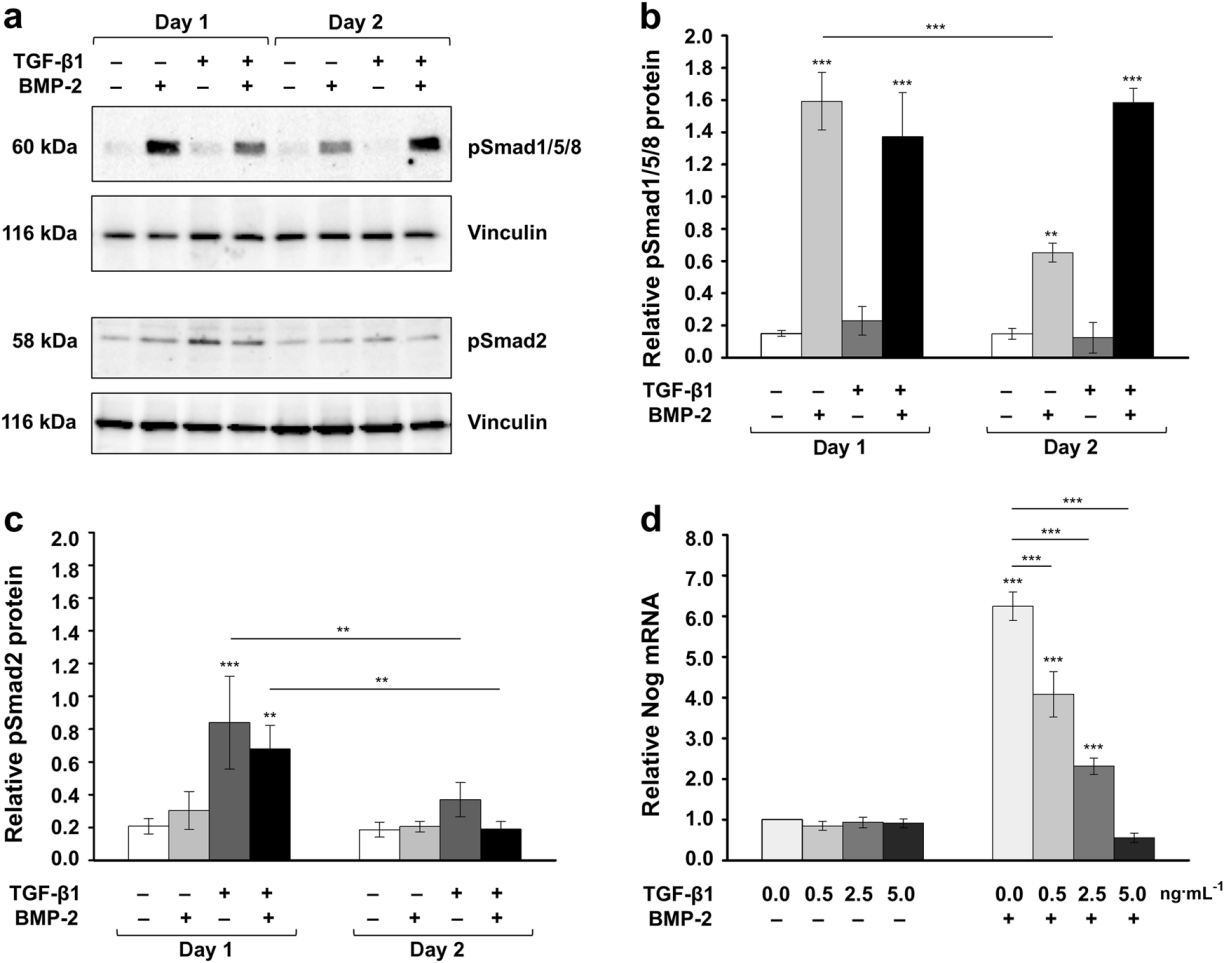
TGF-β1 prolongs BMP-2 signaling and reduces the expression of the BMP-2 antagonist noggin. **a** Immunoblots of phospho-Smad1/5/8 (pSmad1/5/8) and phospho-Smad2 (pSmad2) proteins in whole-cell extracts of ST2 cells that were either untreated or treated with recombinant TGF-β1 (5 ng·ml^−1^), BMP-2 (100 ng·mL^-1^) or both growth factors. Cell lysates were collected on two consecutive days after the treatment. Anti-vinculin served as a loading control. **b**, **c** Densitometric analyses of the immunoblots shown in **a**. pSmad1/5/8 (**b**) and pSmad2 (**c**) protein levels are normalized to the vinculin loading controls. Mean± standard deviations were obtained from three independent experiments, and significant differences to either untreated cells or between identically treated cells on day 1 and 2 after the stimulation (***P < 0.001, **P < 0.01) are shown. **d** Effect of TGF-β1 on BMP-2-induced noggin expression in ST2 cells. Cells were treated with increasing concentrations (0.0, 0.5, 2.5, and 5.0 ng·mL^-1^) of TGF-β1 in the absence (−) or presence (+) of 100 ng·mL^-1^ BMP-2 for 24 h before total RNA was extractedand Nog mRNA levels were analyzed by qRT-PCR. Values normalized to Gapdh are expressed relative to the values of untreated cells. Mean± standard deviations were obtained from three independent experiments, and significant differences to either untreated cells or cells treated with BMP-2 alone (***P < 0.001) are shown

To gain insight into the mechanism determining the stimulatory effect of TGF-β1 on the BMP-2-triggered osteoblast differentiation, we stimulated ST2 cells with TGF-β1 and/or BMP-2 and monitored the levels of phosphorylated Smad1/5/8 and Smad2 proteins (Fig. 5a–c). BMP-2 stimulation resulted in increased active Smad1/5/8 levels after 24 h, which were reduced on day 2 after the treatment (Fig. 5a, b). However, when the cells were co-stimulated with both growth factors, the pSmad1/5/8 levels remained high on day 2, suggesting that TGF-β1 prolongs BMP2 signaling. In contrast, Smad2 phosphorylation was enhanced upon stimulation with TGF-β1 on day 1 but was significantly reduced on day 2, irrespective of the presence or absence of exogenous BMP-2 during the stimulation (Fig. 5a, c).

It is well known that in response to BMP-2, 4, or 6, osteoblasts dramatically upregulate the expression of the secreted glycoprotein noggin, which in turn can limit excessive exposure of cells to BMP signaling through a negative feedback loop.^21^ This prompted us to investigate the effect of TGF-β1 on BMP-2-induced noggin expression in ST2 cells (Fig. 5d). When the two growth factors were applied concomitantly, TGF-β1 significantly reduced the BMP-2-induced Nog mRNA levels in a dose-dependent manner. This might enable prolonged BMP signaling, ultimately contributing to increased osteoblast differentiation.

## Discussion

GBR with a combination of autologous bone chips, DBBM particles, and collagen membranes has consistently shown favorable regenerative outcomes with accelerated bone formation and long-term peri-implant hard and soft tissue stability.^2,7,22,23^ During the augmentation procedure, the surgeon has the viable option to harvest the autologous bone chips locally and store them in a dish containing physiological solution and the patient’s own blood during the time of the surgical implant site preparation, lasting on average for 45 min. Such short-term-extracted BCM can be further used to pre-coat the biomaterials used in the GBR procedure. Thus, the aim of the present study is to extract BCM for the time periods and conditions that are typically encountered in everyday clinical practice and to investigate its activity on the proliferative and osteogenic potential of mesenchymal stromal ST2 cells.

Our data demonstrated a rapid release of TGF-β1 from autologous bone chips within 10 min versus a delayed BMP-2 release from 40 min onwards, suggesting that TGF-β1 and BMP-2 may act both simultaneously and in a sequential manner to promote ST2 cell differentiation towards the osteoblast phenotype. Indeed, we showed that BCMs extracted within short time periods (10, 20, and 40 min), corresponding to the time of a typical surgical procedure, act by increasing ST2 cell proliferation and by inducing Col1 and Spp1 gene expression. These findings agree with in vivo findings, showing that the exogenous delivery of TGF-β enhances bone regeneration around orthopedic implants over a 4-week period with increased regenerated bone volume fraction, bone contact area, and reduced implant-tissue gap,^24^ likely because of the ability of TGF-β to induce the proliferation and recruitment of osteoprogenitors to the implant site. However, depending on the context, inhibitory effects of TGF-β on bone formation have also been reported, e.g., transgenic mice with an osteoblast-specific overexpression of TGF-β2 exhibited an osteoporosis-like phenotype,^25^ whereas, the pharmacological blocking of TGF-β signaling yielded increased bone formation in mature mice.^26^ In our study, we found that BCMs extracted within longer time periods (1, 3, and 6 days), corresponding to the early days after the augmentation procedure, act by promoting the later stages of osteoblast differentiation including matrix mineralization. In contrast, short-term-extracted BCMs, in which TGF-β1 but no BMP-2 was detected, caused a decrease in the expression of the late differentiation markers Bglap2 and Ibsp. As soon as both growth factors were simultaneously present in the BCM, no inhibitory effects on osteoblast differentiation were evident, suggesting a synergistic interplay between TGF-β1 and BMP-2. Consequently, we showed a stimulatory and dose-dependent effect of recombinant TGF-β1 on BMP-2-induced osteoblast differentiation. Thus, besides the well-acknowledged inhibitory effects of TGF-β on the later phases of osteoblast differentiation and maturation,^27–29^ as well as the reported opposite effects of TGF-β and BMP on osteoblast differentiation,^30,31^ our study indicates that TGF-β1 alone as well as in conjunction with BMP-2 can certainly exert positive effects on the osteogenic process. Consistent with our findings, others have shown that TGF-β can enhance BMP-7-induced in vivo bone formation in primates.^32^ Furthermore, it was shown that blocking TGF-β signaling, by means of siRNA-mediated knockdown of its receptors or pharmacological inhibition of ALK5 kinase activity, impedes BMP-6-induced osteoblast differentiation.^33^

Our study further aimed to discover the mechanism by which TGF-β1 exerts its stimulatory effect on the BMP-2-triggered osteoblast differentiation. We found that upon co-stimulation with TGF-β1 and BMP-2, phosphorylated Smad1/5/8 levels in ST2 cells were maintained for a longer period of time, suggesting that TGF-β1 enables prolonged BMP signaling, and thus contributes to the increased osteoblast differentiation. Moreover, we offer a possible explanation for the prolonged BMP signaling through our finding that TGF-β1 significantly inhibited BMP-2-induced noggin transcription. Other signaling molecules such as Sox9, TGF-β3, FGF-2, and -18 are shown to regulate BMP signaling by manipulating noggin expression.^33–36^ By suppressing noggin, endogenously produced BMP agonists may be left unopposed to drive the differentiation of osteoprogenitors, thereby leading to more rapid bone formation and bone defect repair. Given that BCM is enriched in numerous growth factors, we hypothesized that the suppression of noggin by the interplay between simultaneously present growth factors might promote BCM-induced BMP signaling and subsequent osteogenesis.

A clear limitation of the present study is the use of porcine mandibular cortical bone for the preparation of the BCM. Future research is needed to investigate the time-dependent release of growth factors in BCM harvested from autologous bone chips of human origin. Moreover, mass spectrometry-based strategies should be utilized to qualitatively and quantitatively probe human BCM on a proteome scale. Another limitation of the study is the use of a single surgical technique (a bone scraper) to obtain the bone chips needed for the preparation of BCM. BCM harvested from the bone chips obtained using a bone mill, piezo-surgery, or bone drill might have a different composition, and different quantities of paracrine growth factors might be released over time.

Despite the clear limitations that need to be addressed by future research, our data represent a conceptual advance in the understanding of the favorable outcome achieved with the use of BCM and autografts in implant dentistry and point to a novel synergistic relationship between the TGF-β1 and BMP-2 growth factors contained in BCM for the stimulation of osteogenic differentiation. Our ultimate objective is to translate the basic scientific observations into clinical practice. The current study provides strategies towards achieving this goal. First, the mixture of patient blood and RS, in which the autologous bone chips are placed and stored for a minimum of 20 min during surgical implant site preparation, might induce downstream signaling events upon transplantation. Second, BCM obtained during the time of the surgical procedure as well as BCM released from the autograft in situ within 1 day after the augmentation procedure might expand the pool of committed osteoblasts by inducing cell proliferation. Finally, BCM extracted within the time of the surgical procedure has sufficient potency to stimulate the production of bone matrix, whereas BCM released from the autograft in situ after the surgical procedure will continuously contribute to the progression of osteogenesis.

## Materials and methods

### BCM preparation and ELISA protein quantification

BCM was prepared as described.^12^ In brief, cortical bone chips were harvested from the buccal side of fresh pig mandibles (slaughterhouse: Küng Metzgerei, Toffen, Switzerland) using a bone scraper (Hu-Friedy, Rotterdam, The Netherlands) and were placed into either Ringer’s solution (RS) or Ringer’s solution mixed with autologous serum (RS+S) at a 1:1 ratio. A ratio of 5 g bone chips per 10 mL medium was used. Each of the two types of media, abbreviated as BCM-RS and BCM-RS+S, was supplemented with antibiotics and antimycotics. BCM from four independent preparations was collected at 10, 20, and 40 min and at 1, 3, and 6 days, sterile-filtered, and kept frozen at −80 °C.

The release of TGF-β1 or BMP-2 protein in BCM preparations was quantified using Quantikine^®^ colorimetric sandwich ELISA (R&D Systems, Zug, Switzerland), according to the manufacturer’s procedure. Absorbance was measured at 450 and 570 nm using an ELx808 Absorbance Reader (BioTek, Luzern, Switzerland). Data represent mean±SD from three independent experiments performed in duplicate.

### Cell culture and proliferation assay

ST2 mesenchymal stromal cells isolated from mouse bone marrow were obtained from RIKEN Cell Bank (Tsukuba, Japan), and were grown in DMEM medium supplemented with 10% fetal calf serum (FCS; Invitrogen, Zug, Switzerland). For the differentiation experiments, media were supplemented with 50  ascorbic acid (Invitrogen) and 2 mM β-glycerophosphate (Invitrogen), as described.^9^ Cells were treated with either fivefold-diluted BCM or recombinant TGF-β1 and/or BMP-2 proteins (PeproTech, London, UK) for 30 min (protein analyses), 24 h (RNA analyses), or 4 days (mineralization analyses).

Proliferation rates of BCM-treated ST2 cells were determined using a BrdU incorporation assay (Roche, Basel, Switzerland), as described.^37^ In brief, after 24 h of starvation, the cells were plated in triplicate on black 96-well plates (PerkinElmer, Basel, Switzerland) at 2 × 10^3^ cells/well in 3% FCS/DMEM and were allowed to proliferate for 0, 24, 48, 72, and 96 h before labeling with BrdU for 2 h. BrdU incorporation into newly synthesized DNA was determined according to the manufacturer’s protocol using an Infinite® 200 luminometer (Tecan, Männedorf, Switzerland). Experimental values were normalized to the values of ST2 cells treated with RS or RS+S at time point 0. Data represent means±SD from four independent experiments.

### qRT-PCR

Total RNA from BCM- or growth factor-treated ST2 cells was isolated using the RNeasy Mini Kit (Qiagen, Basel, Switzerland). RNA was reverse transcribed, and relative transcript levels for Col1a1, Col1a2, Spp1, Runx2, Bglap2, Ibsp, Alpl, and Nog genes, normalized to Gapdh, were measured using FastStart Universal SYBR Green Master ROX (Roche) and the primer sequences listed in Table 1. qPCR was carried out in a 7500 Real-Time PCR System (Applied Biosystems, Rotkreuz, Switzerland) using a standard thermal cycling profile. Data were analyzed using the efficient ∆∆Ct method.^38^ All samples were run in duplicate. Data represent means±SD from three independent experiments.

**Table 1:** Primer sequences

Gene symbol	Gene bank accession number	Primer pair (fwd/rev)	Amplicon size (bp)
Col1a1	NM_007742.3	5′- CCGGAAGAATACGTATCACCA-3′ 5′- TCTGGGAAGCAAAGTTTCCT-3′	199
Col1a2	NM_007743.3	5′- CTTACTGGGAACTTTGCTGCTC-3′ 5′- TTTCCAGGGTGACCATCCTC-3′	229
Spp1	NM_001204201.1	5′- GGAAACCAGCCAAGGTAAGC-3′ 5′- TGCCAATCTCATGGTCGTAG-3′	92
Runx2	NM_001271627.1	5′- AGGGACTATGGCGTCAAACA-3′ 5′- GGCTCACGTCGCTCATCTT-3′	137
Bglap2	NM_001032298.3	5′- CACCTAGCAGACACCATGAG-3′ 5′- TGGACATGAAGGCTTTGTCAG-3′	123
Ibsp	NM_008318.3	5′- GGTCTTTAAGTACCGGCCAC-3′ 5′- CGTTTGAAGTCTCCTCTTCCTC-3′	157
Alpl	NM_001287172.1	5′- GCAACTCCATCTTTGGTCTG-3′ 5′- GTTGTTGTGAGCGTAATCTACC-3′	145
Nog	NM_008711.2	5′- GCCAGCACTATCTACACATCC-3′ 5′- GCGTCTCGTTCAGATCCTTCTC-3′	114
Gapdh	NM_008084.2	5′-CTTGTGCAGTGCCAGCCTC-3′ 5′-GCCGTGAGTGGAGTCATACTG-3′	189

### Immunoblotting

Whole-cell extracts from BCM- or growth factor-treated ST2 cells were prepared by lysis in RIPA buffer (50 mM Tris-HCl, pH 7.4, 150 mmol·L^-1^ NaCl, 1% NP-40, 0.25% sodium deoxycholate, 1 mmol·L^-1^ EDTA, 1 × complete Protease Inhibitor Cocktail (Roche)), as described.^39^ Samples were run on 10% SDS–PAGE and transferred to Protran® membranes (Sigma, Buchs, Switzerland). Proteins of interest were visualized using anti-phospho-Smad1/5/8 (Cell Signaling Technology, Leiden, The Netherlands), anti-phospho-Smad2 (ThermoFisher Scientific, Reinach, Switzerland), and anti-vinculin (Sigma) antibodies, followed by horseradish peroxidase-conjugated secondary antibodies for detection with the SuperSignal™ West Dura Substrate (ThermoFisher Scientific). Phospho-Smad1/5/8 or phospho-Smad2 protein expression, relative to the vinculin loading control, was quantified by densitometry using ImageQuant Software (Molecular Dynamics Inc., Sunnyvale, CA, USA). Data represent mean±SD from three independent experiments.

### Alizarin red staining

Mineral deposition of BCM- or growth factor-treated ST2 cells was analyzed by alizarin red staining after 14 days using 0.2% Alizarin Red S (Sigma), pH 6.4. Images were acquired on an Olympus BX-51. Mineralization was quantified by measuring the stained area using the Fiji distribution of ImageJ.^40^ Cellular DNA content, as measured by the CyQUANT^®^ NF assay (Invitrogen), was used for normalization. Data represent mean±SD from three independent experiments.

### Statistical analysis

All grouped data are mean±SD. Differences between the groups were assessed by one-way analysis of variance (ANOVA) using GraphPad InStat Software, version 3.05. Values of *P* < 0.05 were considered significant.

### Data availability statement

Availability of data and materials should be included here.
